# Functional Fallout: Short-Term Outcomes Following Hip Fracture in Patients Aged 90 and Over at a United Kingdom District General Hospital

**DOI:** 10.7759/cureus.88310

**Published:** 2025-07-19

**Authors:** Hashim Al-Hano, Molly Handcock

**Affiliations:** 1 Department of General Surgery, Royal Hampshire County Hospital, Winchester, GBR

**Keywords:** discharge planning, elderly trauma, functional decline, geriatric orthopaedics, hip fracture, institutionalisation, mobility outcomes, nonagenarians, orthogeriatric care, postoperative recovery

## Abstract

Introduction

Hip fractures in patients aged ≥90 years pose distinct clinical and functional challenges due to advanced frailty and limited physiological reserve. While long-term outcomes are well documented in the elderly, short-term discharge-related functional outcomes remain underexplored in nonagenarians. This study evaluated post-operative mobility decline, discharge needs, and early mortality in this high-risk cohort.

Methods

We retrospectively reviewed 84 patients aged ≥90 years who underwent surgical fixation for radiologically confirmed hip fractures between August 2024 and April 2025 at a United Kingdom District General Hospital. Demographics, American Society of Anaesthesiologists (ASA) grade, fracture type, operative method, mobility (pre- and post-operative), discharge destination, length of stay, return to theatre, and 30-day mortality were analysed. Functional decline was defined as a drop in ordinal mobility score from baseline to discharge. Institutionalisation was defined as discharge to a care facility (rehabilitation, residential home, or nursing home) due to a decline in functional mobility.

Results

The mean age was 92.9 years (range 90-102); 69% were female. Intracapsular fractures comprised 57% of cases. Most patients (84.5%) were ASA grade 3. Hemiarthroplasty was the most common procedure (59.5%), followed by dynamic hip screws (21.4%), nail fixation (17.9%), and total hip replacement (1.2%). Functional mobility declined in 78% of patients and remained unchanged in 21%. Only one patient (1.2%) retained full independence. At discharge, 25% used a frame independently, 37% required one-person (AO1), and 16% required two-person (AO2) assistance. Thirty-day mortality was 8.3%; return to theatre occurred in 4.8%. Mean length of stay was 18.2 days. Discharge destinations included home (32%), rehab (26%), residential home (19%), and nursing home (13%). Institutionalisation due to mobility decline occurred in 41% of patients. Patients previously independent pre-fracture experienced the greatest average decline in mobility score (-2.35).

Conclusion

Hip fracture in nonagenarians is associated with high rates of early functional decline and institutionalisation. Despite established multidisciplinary care, outcomes suggest the need for age-specific discharge planning, tailored rehabilitation, and proactive functional risk stratification to support recovery.

## Introduction

Hip fractures in patients aged ≥90 years present unique clinical and logistical challenges due to advanced frailty, limited physiological reserve, and often high baseline care needs. While long-term mortality and mobility outcomes following hip fractures have been well described in older adults [1], there is limited literature detailing immediate functional status at discharge in this very elderly subgroup. This study aims to provide a real-world assessment of post-operative functional decline, care transitions, and early mortality in nonagenarians following surgical management of hip fractures at a UK District General Hospital.

## Materials and methods

Study design

We performed a retrospective review of 84 patients aged 90 years and older who underwent surgical treatment for radiologically confirmed hip fractures between August 2024 and April 2025.

Inclusion criteria

All patients aged ≥90 years admitted with radiologically confirmed proximal femoral fractures who received operative management.

Exclusion criteria

Patients managed non-operatively or with pathological fractures (e.g., secondary to malignancy) were excluded.

Data collection

Demographic data, ASA grade, fracture type (intracapsular or extracapsular), surgical procedure (hemiarthroplasty, total hip replacement (THR), dynamic hip screw (DHS), or intramedullary nail), pre- and post-operative mobility, discharge destination, length of hospital stay, 30-day mortality, and return to theatre were collected from electronic medical records.

Mobility scoring and definitions

Pre- and post-operative mobility were converted into ordinal scores on a 0-5 scale, with higher scores indicating greater independence. This allowed quantitative comparison of functional decline. Discharge destination was categorized as home, rehabilitation, residential home, or nursing home. Institutionalisation was defined as discharge to a more dependent setting than pre-fracture residence.

Data analysis

Descriptive statistics were used to summarise variables. Subgroup comparisons were made for mobility decline by fracture type, surgical procedure, and pre-fracture mobility level. Inferential testing was not performed due to limited sample size; this limitation is acknowledged in the discussion.

Subgroup analysis

Subgroup analysis was performed to evaluate the average change in mobility scores by fracture type and surgical procedure. Mobility scores were averaged within each group, and mean differences were compared to identify trends in functional decline associated with treatment type or fracture classification.

Ethical approval

This study was registered as a local service evaluation (Ref: HHFT-SE-2024-0021). Ethics committee approval was not required in accordance with HRA guidelines.

## Results

The mean age of the cohort was 92.9 years (range 90-102), with 69% (n=58) female and 31% (n=26) male (Table 1). The majority were ASA grade 3 (84.5%, n=71), with ASA grade 2 comprising 4.8% (n=4) and ASA grade 4 accounting for 10.7% (n=9). Pre-fracture residence was predominantly at home (69%, n=58), followed by residential care homes (20%, n=17) and nursing homes (11%, n=9).

**Table 1:** Summary of demographics and outcomes. ASA: American Society of Anesthesiologists; DHS: Dynamic Hip Screw; THR: Total Hip Replacement.

Variable	Value
Total patients	84
Mean age (years)	92.9 (range 90-102)
Female : Male	58 (69%) : 26 (31%)
ASA grade 3	71 (84.5%)
ASA grade 4	9 (10.7%)
ASA grade 2	4 (4.8%)
Intracapsular fractures	48 (57%)
Extracapsular fractures	36 (43%)
Hemiarthroplasty	50 (59.5%)
Dynamic hip screw (DHS)	18 (21.4%)
Intramedullary nail	15 (17.9%)
Total hip replacement (THR)	1 (1.2%)
Functional mobility decline	65 (78%)
30-day mortality	7 (8.3%)
Return to theatre	4 (4.8%)
Mean hospital stay (days)	18.2 (median 13, range 3-48)
Discharged to more dependent care	34 (41%)

Fracture types included intracapsular (57%, n=48) and extracapsular (43%, n=36). Surgical procedures performed were hemiarthroplasty (59.5%, n=50), DHS fixation (21.4%, n=18), intramedullary nail fixation (17.9%, n=15), and THR (1.2%, n=1).

Mobility at discharge declined in 78% (n=65) of patients, remained unchanged in 21% (n=18), and improved in 0%. Only one patient (1.2%) retained full independence. At discharge, 25% (n=21) were using a frame independently, 37% (n=31) required one-person assistance with a frame (AO1), and 16% (n=13) required two-person assistance (AO2). The remaining 22% (n=19) included patients who were bed-bound or using other mobility aids with more significant support (e.g., hoist transfer).

Return to theatre occurred in four patients (4.8%), with indications including infection (n=3) and mechanical failure (n=1). Thirty-day mortality was 8.3% (n=7). The mean hospital length of stay was 18.2 days, with a median of 13 days (range 3-48).

Discharge destinations were as follows: home (32%, n=27), rehabilitation unit (26%, n=22), residential home (19%, n=16), and nursing home (13%, n=11). The remaining 10% (n=8) either died during admission or were discharged to a hospice or palliative care setting. Overall, 41% (n=34) were discharged to more dependent settings than their pre-fracture residence.

Patients with intracapsular fractures had a mean mobility score decline of -1.42, compared to -1.72 for those with extracapsular fractures, indicating moderately better outcomes. Similarly, patients who underwent hemiarthroplasty showed the least functional decline on average (-1.42), followed by those who had a THR (-2.0), DHS fixation (-1.67), and nail fixation (-1.80).

In terms of mobility status decline, those who were previously independent experienced an average decline of -2.35, compared to -1.67 for those using a stick, -1.13 for those using a frame, and -0.33 or less for patients who were chair- or bed-bound pre-fracture.

## Discussion

Our findings highlight the marked short-term functional decline following hip fracture in patients aged ≥90 years, despite standard early mobilisation and multidisciplinary care pathways. Standard early mobilisation included a physiotherapist review on post-operative day 1 with a trial of mobilisation using the patient’s baseline mobility. For example, if the patient was mobilising with a stick pre-fracture, then a stick would be trialled initially. Mobilisation trials continued daily for the first three days, then every other day for the remainder of the hospital stay. The regimen included short walks (about 10 meters), progressing to longer walks and eventually stair assessments. Although the mean length of stay was comparable to that in broader elderly populations [2], discharge to dependent care settings occurred more frequently. Notably, 78% of patients experienced a decline in mobility, and 41% were institutionalised, compared to institutionalisation rates of around 30% in the general elderly population [3]. In these patients, institutionalisation was necessary to ensure safe discharge or provide further rehabilitation.

Intracapsular fractures and hemiarthroplasty were associated with marginally better functional outcomes, consistent with findings from the National Hip Fracture Database and broader geriatric orthopaedic literature [4]. Intracapsular fractures appear to be less disruptive to surrounding soft tissues and generally do not involve the greater trochanter or abductor mechanism (though this may be disrupted depending on the surgical approach). Arthroplasty bypasses the need for fracture healing and provides immediate mechanical stability, facilitating earlier weight-bearing and mobilisation.

Pre-fracture independence predicted greater absolute loss in mobility. This apparent paradox may reflect the greater potential for functional decline in previously independent patients. These findings support the development of risk models incorporating ASA grade, age, and baseline function to inform early discharge planning.

While many best-practice interventions are already embedded in care pathways (e.g., early mobilisation, orthogeriatric review), our data suggest that these protocols may need to be further individualised for very elderly patients. Tailored rehabilitation programs and discharge planning tools specific to nonagenarians may improve resource allocation and care continuity [5]. Future work may explore predictive modelling and prospective validation.

## Conclusions

Hip fractures in nonagenarians lead to significant short-term functional decline and increased care dependency. Despite established multidisciplinary care pathways, outcomes remain suboptimal. Incorporating risk stratification tools and individualized discharge planning may support recovery and help reduce institutionalisation in this high-risk population.
